# Multiple imputation of systematically missing data on gait speed in the Swedish National Study on Aging and Care

**DOI:** 10.18632/aging.205552

**Published:** 2024-02-14

**Authors:** Robert Thiesmeier, Ahmad Abbadi, Debora Rizzuto, Amaia Calderón-Larrañaga, Scott M. Hofer, Nicola Orsini

**Affiliations:** 1 Department of Global Public Health, Karolinska Institutet, Stockholm, Sweden; 2 Aging Research Center, Department of Neurobiology, Care Sciences and Society, Karolinska Institutet, and Stockholm University, Stockholm, Sweden; 3 Stockholm Gerontology Research Center, Stockholm, Sweden; 4 Department of Neurology, Oregon Health and Science University, Portland, OR 97239, USA

**Keywords:** simulation, systematically missing values, individual participant data, meta-analysis, gait speed

## Abstract

Background: There is insufficient investigation of multiple imputation for systematically missing discrete variables in individual participant data meta-analysis (IPDMA) with a small number of included studies. Therefore, this study aims to evaluate the performance of three multiple imputation strategies – fully conditional specification (FCS), multivariate normal (MVN), conditional quantile imputation (CQI) – on systematically missing data on gait speed in the Swedish National Study on Aging and Care (SNAC).

Methods: In total, 1 000 IPDMA were simulated with four prospective cohort studies based on the characteristics of the SNAC. The three multiple imputation strategies were analysed with a two-stage common-effect multivariable logistic model targeting the effect of three levels of gait speed (100% missing in one study) on 5-years mortality with common odds ratios set to *OR*_1_ = 0.55 (0.8-1.2 vs ≤0.8 m/s), and *OR*_2_ = 0.29 (>1.2 vs ≤0.8 m/s).

Results: The average combined estimate for the mortality odds ratio *OR*_1_ (relative bias %) were 0.58 (8.2%), 0.58 (7.5%), and 0.55 (0.7%) for the FCS, MVN, and CQI, respectively. The average combined estimate for the mortality odds ratio *OR*_2_ (relative bias %) were 0.30 (2.5%), 0.33 (10.0%), and 0.29 (0.9%) for the FCS, MVN, and CQI respectively.

Conclusions: In our simulations of an IPDMA based on the SNAC where gait speed data was systematically missing in one study, all three imputation methods performed relatively well. The smallest bias was found for the CQI approach.

## INTRODUCTION

The development of statistical models that diagnose and predict the occurrence of disease outcomes is pivotal to inform clinical diagnosis and prognosis [1]. Such multivariable prediction models are increasingly built with individual participant data meta-analyses (IPDMA) [2]. Despite its benefits, using individual participant data often comes at the cost of introducing practical and methodological challenges, [3] such as systemically missing data [4]. Systematically missing data occurs when a variable is not measured in one or more included studies – often due to difference in survey instruments, measurement devices, or inadequate information [5, 6]. Such missing data poses a pivotal challenge for IPDMA in clinical and epidemiological research [5].

Methodological research is gradually advancing to address these challenges and provide substantiated recommendations across diverse scenarios. Researchers must weigh the trade-offs between losing power and information by excluding studies and using methods like multiple imputation (MI) to estimate missing values based on observed data [2, 6].

The Swedish National Study on Aging and Care (SNAC) is an example of four observational studies (i.e., four studies sites) using individual participant data, thus can be combined for an IPDMA. With over 8,000 participants across the four cohorts, SNAC has facilitated the development of an innovative Health Assessment Tool that integrates indicators of both clinical and functional health in a population aged 60+ years [7]. However, within SNAC, one variable, gait speed, is systematically missing in one study. Researchers must then decide between i) proceeding with complete data using only three studies, thus risking information loss and potential bias in combined estimates [4, 5], or ii) employing Multiple Imputation (MI), which could offer plausible values for the absent study based on observed data. If choosing option ii, selecting the most suitable MI approach becomes crucial, given the limited number of studies in the IDPMA [8]. Although the performances of MI methods for multilevel data with systematically missing data have been evaluated in previous simulation studies covering several scenarios (quantitative and binary predictors [2], magnitude of heterogeneity across studies [9]), few studies have evaluated different MI approaches with only a small number of included studies [2, 8]. A comprehensive overview of MI techniques in the context of systematically missing values in IPDMA has been documented by Audigier et al. [8]. Among one of the main challenges of dealing with systematically missing data in IPDMA is to preserve the structure and relationships within each study [6, 10]. In addition, the majority of MI methods are extensively tested for continuous variables, yet few studies have applied such methods to discrete data.

The systematically missing values for gait speed data in one of the SNAC sites poses a unique methodological challenge in IPDMA involving four larger cohort studies. Consequently, there is a notable gap in identifying suitable MI methods for systematically missing discrete data with only a small number of included studies.

Therefore, this study aims to investigate and assess the performance of different MI strategies specifically targeting the systematically missing discrete variable of gait speed in the SNAC IPDMA with only four large cohort studies.

The remaining part of the paper is organised as follows I) a description of the data sources used to inform the simulation study; II) a presentation of the rationale of two popular imputation methods and one approach based on conditional quantiles to address systematic missingness; III) the design of the simulation study and mechanisms underlying the data; IV) reporting the results of the simulations; and V) a discussion of strengths and limitations of this paper.

## MATERIALS AND METHODS

### Study population and study variables

This simulation study is grounded in data from SNAC, which is an ongoing longitudinal cohort study based on samples of the Swedish elderly population launched in 2001. A detailed description of the study structure and methods can be found elsewhere [11]. In brief, it consists of four study sites, namely Kungsholmen, Skåne, Nordanstig, and Blekinge. Data collection includes information on health determinants, disease outcomes, functional capacity, and social conditions [12]. A key predictor of all-cause mortality – gait speed (≤0.8, 0.8-1.2, >1.2 meters per second) – is systematically missing at one study site, Blekinge. To evaluate potential imputation methods to impute the systematically missing variable we defined the outcome as all-cause mortality (yes/no) within 5 years from the examination date. Further, four key health indicators were chosen based on previous analysis [7] that included, severe disability (measured as the number of personal activities of daily living (ADL) a person was unable to perform independently, categorised into 0 and ≥1), mild disability (measured as the number of instrumental activities of daily living (IADL), categorised into 0 and ≥1), cognitive status measured with the Mini-Mental State Examination (MMSE) ranging from 30 (best possible score) to 0, categorised into ≤20, 20-25 and >25), and the number of chronic diseases (count of chronic diseases performed by a clinical examination (ICD-10 diagnostic criteria); categorised into ≤2, 2-4, >4 chronic diseases). Two demographic factors were also included: sex (female/male), and age (59-70, 70-80, 80-90, 90+ years). The cut-offs were chosen based on a combination of avoiding numerical problems during the simulations (i.e., having a sufficient number of subjects in each category of the variables) and on clinical cut-offs used in Santoni et al. [7].

The following sections outline the structure of the simulation study, including a) the data generating mechanism, b) imputation methods, c) analytical methods, and d) estimands and performance measures [13].

### Data generating mechanism

In this paragraph we describe how we simulated one IPDMA. We created four synthetic data [14] sets based on the original data from the four SNAC sites (Kungsholmen, Skåne, Nordanstig, Blekinge), keeping their main statistical properties. The synthetic data sets included the five above mentioned predictors of 5-years mortality (gait speed, ADL, IADL, MMSE, number of comorbidities) and two demographic factors (sex and age).

Our simulation strategy consisted of first reproducing marginal and conditional relationships of all the predictors separately within each study; and second, generating individual binary outcomes according to a common set of regression coefficients across all four studies.

All predictors of mortality were randomly generated from a multivariate normal distribution given a set of observed means and variance/covariances. The variables were discretised using the inverse cumulative distribution function method based on observed frequencies [15, 16]. To simulate gait speed in the study with missing information (Blekinge), we used the inverse cumulative distribution function method based on an arithmetic average of observed frequencies available in the other studies (Kungsholmen, Skåne, Nordanstig).

We denote with *i* the index for the studies included in the prospective MA data. In our simulated scenario, the index *i* ranges from 1 to 4 representing the Kungsholmen, Skåne, Nordanstig, and Blekinge studies, respectively. Data on seven possibly correlated predictors *X* (gait speed, ADL, IADL, MMSE, comorbidities, age, and sex) of 5-year mortality risk were randomly generated from single multivariate normal distribution [15]


$$X_{i}\sim N(\mu_{i},\Sigma_{i})$$

where Σ*_i_* is the symmetric observed variance/covariance matrix and *μ_i_* is the observed vector of means for the *i*-th study. The variance covariance matrices are based on the real data with the same number of discretised variables (eAppendix A in the Supplement). Next, each variable in *X_i_* was discretised using the inverse of the normal cumulative distribution given the empirical mean *μ_i_*, its standard deviation (square root of the diagonal elements of Σ*_i_*), and the observed probabilities shown in Table 1 of the original data [15, 16].

**Table 1:** Descriptive population characteristics for the key variables of the four studies (Kungsholmen, Skåne, Nordanstieg and Blekinge) from the Swedish National Study on Aging and Care.

	**Kungsholmen**	**Skåne**	**Nordanstieg**	**Blekinge**
**Variables** (predictors)	n=3,363 %	n=768 %	n=2,397 %	n=1,402 %
**Gait speed** (m/s)				
≤0.8	30	33	6	Missing
0.8-1.2	21	46	26	Missing
>1.2	49	21	68	Missing
**ADL**				
≥1 vs 0	10	8	5	13
**IADL**				
≥1 vs 0	24	28	24	44
**MMSE**				
≤20	7	8	3	11
20-25	7	6	19	18
>25	86	86	78	71
**Comorbidities**				
≤2	30	64	17	50
2-4	33	24	36	30
>4	37	12	47	20
**5-years mortality**	22	27	12	26
**Female**	65	54	54	58
Age (years)				
59-70	39	34	54	28
70-80	28	25	20	25
80-90	19	32	22	36
≥90	14	9	4	11

Column percentages (%) are reported.

Legend: Variables included gait speed (in meters per second (m/s)), severe disability (ADL), mild disability (IADL), cognitive status measured with the Mini-Mental State Examination (MMSE), number of chronic diseases (comorbidities), all-cause mortality (5-years mortality), sex (female), and age (in years).

The individual binary outcome, 5-year mortality status, was randomly generated according to a Bernoulli distribution. The outcome probability varied conditionally on all the predictors modelled with indicator variables and a common set of regression coefficients. Independent observations within each study were described by the statistical model *Y_i_*|*X_i_* ~ Bernoulli(*π_i_*) and $\pi_{i}=e^{X_{i}\beta }\text{/}(1+e^{X_{i}\beta })$ with *X_i_β*, the linear predictor of the logit (log odds) of the 5-years mortality probability:


$$\begin{array}{c} X_{i}\beta =-0.786-0.599\;\text{I}(\text{gaitspeed}=2)\;\\ -1.237\text{ I}(\text{gaitspeed}=3)\\ -0.791\text{ I}(\text{MMSE}=2)-1.13\text{ I}(\text{MMSE}=3)\\ +\;0.297(\text{ADL})+0.520(\text{IADL})\\ -0.0197\text{ I}(\text{Cormobidities}=2)\\ \;+\;0.297\text{ I}(\text{Comorbidities}=3)\\ -0.725(\text{Sex})+0.949\;\text{I}(\text{Age}=2)\\ +\;1.382\text{ I}(\text{Age}=3)\\ +\;2.17\text{ I}(\text{Age}=4) \end{array}$$

The values of the regression coefficients above were obtained by computing the inverse-variance weighted average of the regression coefficients estimated in the three studies with complete data. The common adjusted effects of gait speed on 5-year mortality risk comparing 0.8-1.2 vs ≤0.8 m/s was $OR_{1}=e^{\beta_{1}}=e^{-0.599}=0.55$ and comparing >1.2 vs ≤0.8 m/s was $OR_{2}=e^{\beta_{2}}=e^{-1.237}=0.29$. These parameter values served as a benchmark to evaluate the performance of the different imputation strategies. Once the individual mortality outcomes were generated based on the above realistic values of the regression coefficients and data, we set gait speed to systematically missing in the study SNAC-Blekinge. Each of the three imputation strategies described in the following paragraph were then used for the same IPDMA to impute gait speed.

### Imputation methods

This paragraph describes the three evaluated imputation methods. Based on an IPDMA of four studies, we evaluated two standard imputation methods of systematically missing discrete data, fully conditional specification (FCS) and multivariate normal (MVN). In addition, we evaluate a method based on conditional quantiles (CQI). We imputed the systematically missing variable 100 times for each imputation method given that the Monte Carlo Error (MCE) for 100 imputations and 1 000 repetitions is expected to be very small [17].

### Fully conditional specification (FCS)

FCS was first described in detail by van Buuren, Boshuizen and Knook [18]. It identifies a suitable conditional imputation model for each incomplete variable and iteratively imputes until convergence [10, 17–19]. We used FCS for one systematic missing discrete variable. As an imputation model for the discrete missing variable gait speed, we used multinomial logistic regression on joined (appended) datasets [20]. The model included all of the previously mentioned predictors: ADL, IADL, MMSE, number of comorbidities, sex, age, mortality outcome, and study-level indicator variables to identify the original structure of the data.

### Multivariate normal (MVN)

MVN imputation assumes that variables being imputed follow a multivariate normal distribution [21]. The method uses an iterative Markov Chain Monte Carlo method to impute missing values [22]. The performance of MVN has also been investigated and evaluated for binary variables [23–25]. The MVN imputation model for gait speed included the same predictors as mentioned for the FCS.

### Conditional quantile imputation

This paragraph provides a brief description of a conditional quantile imputation (CQI) approach [26]. A more detailed description of the imputation method can be found in Bottai et al. [27]. Based in Bottai et al. [27] the rationale of the imputation method consists of three main steps: I) quantification of the association between the missing variable, gait speed in our example, and any other observed variable in the three studies (Kungsholmen, Skåne, Nordanstig) where the missing variable is available; II) prediction of the probabilities of any level of the discrete missing variable conditionally on the observed variables in the study with missing data (Blekinge) based on the estimated average relationships obtained in the previous step; and III) imputation of individual missing values of the discrete variable by inverting the cumulative distribution function of a random uniform with quantiles equal to the cumulative predicted conditional probabilities. More technical details of the steps involved in CQI are noted in eAppendix B in the Supplement of this paper.

### Analytical methods

The results of the imputation methods were analysed with the following analytical model. MI methods that heavily rely on random-effect models in the case of limited number of studies are difficult to estimate [8]. Moreover, the harmonisation in measurement and data collection between the four study sites of SNAC allow us to assume a common effect as opposed to a heterogenous (random) effect. Thus, the multivariable logistic model described above to predict 5-years mortality risk was estimated for any simulated IPDMA using a two-stage common effect meta-analysis. Estimates of the regression coefficients were combined across imputations using Rubin’s rules [28].

### Simulation estimands and performance measure

We simulated the mechanism described above 1 000 times to obtain a sampling distribution of the adjusted effect of gait speed, the predictor that is systematically missing, on mortality risk. Gait speed was modelled with two indicator variables. The performance of the three imputation methods was assessed for the two corresponding regression coefficients (the conditional log odds ratios) of gait speed. The key numerical quantity used to assess the performance was the average relative bias comparing the estimated regression coefficients $\left({\hat{\beta }}_{1},{\hat{\beta }}_{2}\right)$ with the parameter values in the outcome model previously specified (*β*_1_ = −0.599 and *β*_2_ = −1.237). In addition, we estimated the following performance measures including their Monte Carlo Error (MCE) described in Morris et al. [13] for all three methods: i) bias in point estimate, ii) model-based standard error (SE) (the mean of the SE from the 1 000 repetitions), iii) empirical SE (the standard deviation of the 1 000 estimates from the 1 000 repetitions), and iv) nominal coverage level (proportion of CIs covering the reference value).

### Application

We applied the three imputation methods to the original SNAC data. In addition to the adjusted odds ratio and 95% confidence interval, we calculated the predictive capacity of the model based on the area under the curve (AUC) [29]. The imputation and analytical strategy followed the same procedure as in the simulations.

### Availability of data and materials

The datasets used and/or analysed during the current study are available from the corresponding author on reasonable request.

## RESULTS

Table 1 shows the empirical frequency distribution (%) of all variables of the four original studies. The 5-years mortality risk ranged from 12% to 27% across the four studies. The distribution of gait speed also varied across the studies. In particular, the fraction of individuals with a gait speed above 1.2 m/s was 21%, 49%, and 68% in the Kungsholmen, Skåne, and Nordanstig studies, respectively.

### Simulation results

Table 2 describes the combined adjusted estimates of the levels of gait speed on 5-years mortality and performance measures for the three imputation methods based on 1 000 simulations. We used the first level of gait speed as a reference (≤0.8 m/s). The average combined estimate for the mortality odds ratio and the relative bias (%) for the second level of gait speed (0.8-1.2 vs ≤0.8 m/s) were highest for the FCS method ${\hat{OR}}_{1}=0.579$; relative bias = 8.2%), and lowest for the CQI method (${\hat{OR}}_{1}=0.55$; relative bias = 0.7%). For the third level of gait speed (>1.2 vs ≤0.8 m/s), estimates were highest for the MVN method (${\hat{OR}}_{2}=0.33$; relative bias = 9.9%). Compared to FCS and MVN, CQI seems less efficient due what can be seen in a higher empirical SE (0.096 compared to 0.074 and 0.079 for MVN and FCS, respectively. The fraction of simulated studies in which the parameter values were included in the confidence intervals were 95.80%, 96.70%, and 94.90% for FCS, MVN, and CQI, respectively.

**Table 2:** Combined adjusted effect estimates (log odds ratios) $({\hat{\beta }}_{1},\;{\hat{\beta }}_{2})$ of the non-reference levels of gait speed (0.8-1.2 vs ≤ 0.8 m/s and >1.2 vs ≤ 0.8 m/s) on 5-years mortality for three multiple imputation (MI) methods based on 1 000 simulations.

	***FCS***	***MVN***	***CQI***
**Gait speed 0.8-1.2 vs ≤ 0.8 m/s**	**Estimate**	**Estimate**	**Estimate**
Average ${\hat{\beta }}_{1}$	-0.550	-0.555	-0.604
Average $\hat{SE(\beta_{1})}$	0.095	0.092	0.096
Bias in point estimate	0.049	0.045	-0.004
Relative bias (%)	8.236	7.484	0.711
Empirical SE	0.079	0.074	0.097
Nominal coverage (MCSE)	95.80 (0.634)	96.70 (0.565)	94.90 (0.696)
**Gait speed >1.2 vs ≤ 0.8 m/s**			
Average ${\hat{\beta }}_{1}$	-1.205	-1.113	-1.243
Average $\hat{SE(\beta_{1})}$	0.095	0.094	0.096
Bias in point estimate	0.032	0.124	-0.006
Relative bias (%)	2.551	9.994	0.507
Empirical SE	0.091	0.083	0.093
Nominal coverage (MCSE)	94.80 (0.702)	77.10 (1.329)	95.50 (0.656)

Included performance measures are the estimated standard error (Model-based SE) $\left(\hat{SE(\beta_{1})},\;\hat{SE(\beta_{2})}\right)$, bias in point estimate, relative bias, empirical SE, and coverage of nominal 95% confidence interval. The reference parameters are *β*_1_ = −0.599, *β*_2_ = −1.237. Monte Carlo Error (MCE) was below ≤0.003 for all performance measures expect if indicated differently.

Legend: The compared MI methods are fully conditional specification (FCS), multivariate normal (MVN), and conditional quantile imputation (CQI). Each simulation is based on 100 imputations including four synthetic data sets based on the Swedish National Study on Aging and Care. Adjustments were made for severe disability (ADL), mild disability (IADL), cognitive status measured with the Mini-Mental State Examination (MMSE), number of chronic diseases (comorbidities), all-cause mortality (5-years mortality), sex (female), and age (in years).

For the third level of gait speed, the FCS method had a lower relative bias and more precise point estimate compared to its performance for the second level of gait speed (${\hat{OR}}_{2}=0.30$; relative bias = 2.5%). Again, the lowest bias was shown for the CQI method with an average combined estimate for the mortality odds ratio of ${\hat{OR}}_{2}=0.29$ and a relative bias of 0.9%. The average estimated standard error of the combined estimates was similar across all three methods. A slightly higher average estimated spread could be found for the CQI method for the second and third level of gait speed (SE = 0.096). Similar to the second level of gait speed, CQI indicates a less efficient performance when comparing empirical SE. The nominal coverage seems sufficient for FCS and CQI, however is only at 77.10% for MVN. This is not surprising however, given the large bias in point estimate (0.124).

**Figure 1:**
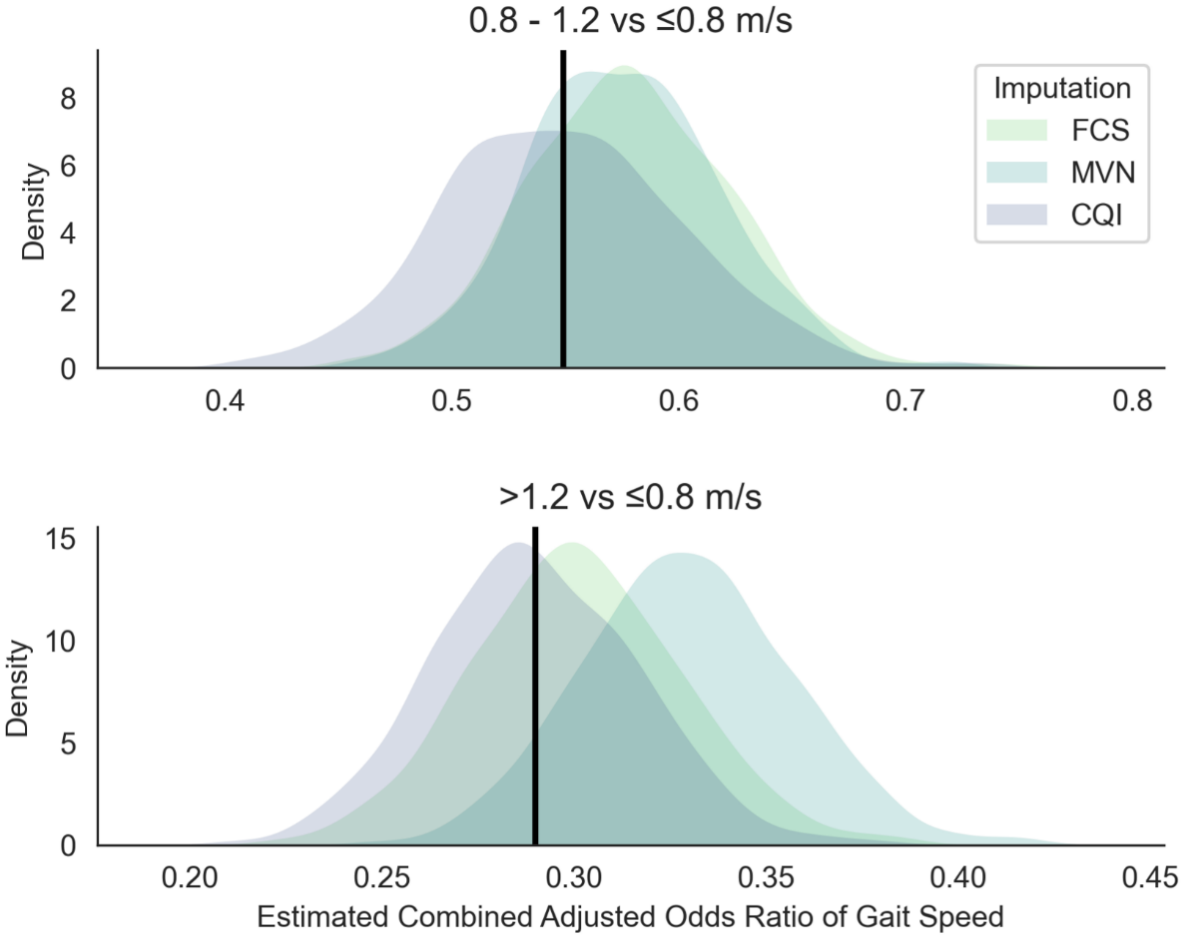
**Simulated sampling distribution of the combined adjusted odds ratio of gait speed on 5-years mortality.** The black line represents the combined adjusted effect *OR*_1_ = 0.55 for the second level of gait speed (0.8-1.2 vs ≤ 0.8 m/s), and *OR*_2_ = 0.29 for the third level of gait speed (>1.2 vs ≤ 0.8 m/s). The three imputation methods that are compared are the fully conditional specification (FCS), multivariate normal (MVN), and conditional quantile imputation (CQI). Simulations are based on 1 000 replications with 100 imputations including four synthetic studies based on the Swedish National Study on Aging and Care. Adjustments were made for severe disability (ADL), mild disability (IADL), cognitive functional status measured with the Mini-Mental State Examination (MMSE), number of chronic diseases (comorbidities), all-cause mortality (5-years mortality), sex (female), and age (in years).

Figure 1 shows the approximately symmetric and bell-shaped simulated distribution of the combined adjusted effect estimates of the three levels of gait speed on 5-years mortality. For comparing the second vs first level of gait speed (0.8-1.2 vs ≤ 0.8 m/s), all three methods share a similar distribution with a substantial overlap. CQI method indicates a better precision and is centred around the estimated common effect of gait speed on mortality (*OR* = 0.548). Both the FCS and MVN share a particularly similar distribution with less spread compared to the CQI method. The effect estimates for the MVN method show a larger divergence in distribution from the estimated common effect of gait speed on mortality for both levels of gait speed (0.8-1.2 vs ≤ 0.8 m/s and >1.2 vs ≤ 0.8 m/s).

### Application to SNAC data

We applied the investigated MI methods to the four original data sets of the SNAC studies where gait speed was systematically missing at the study site in Blekinge. Table 3 shows that the three MI approaches show comparable effect sizes. As expected from the simulation results, there are no large differences between the three methods on the adjusted effect estimates of gait speed on 5-years mortality. For the second level of gait speed (0.8-1.2 vs ≤ 0.8 m/s) the odds ratios for the FCS, MVN, and CQI method are 0.571, 0.568, and 0.563, respectively. In addition, for the third level of gait speed (>1.2 vs ≤ 0.8 m/s) the odds ratios for the FCS, MVN, and CQI method are 0.309, 0.318, and 0.301, respectively. All three methods share a similar predictive capacity around an AUC of 0.82 with CQI being slightly higher compared to FCS and MVN. There was no substantial difference between the MI methods and the complete case analysis including only the three complete data sets.

**Table 3:** Application of three multiple imputation methods to the Swedish National Study on Aging and Care.

	***FCS***	***MVN***	***CQI***	***CC***
**Area under the curve**	0.842	0.841	0.843	0.840
**Gait speed 0.8-1.2 vs ≤ 0.8 m/s**	**Estimate**	**Estimate**	**Estimate**	**Estimate**
Adjusted Odds Ratio	0.571	0.581	0.563	0.549
95% CI	0.465, 0.704	0.472, 0.715	0.456, 0.689	0.445, 0.678
**Gait speed >1.2 vs ≤ 0.8 m/s**				
Adjusted Odds Ratio	0.309	0.332	0.301	0.290
95% CI	0.242, 0.397	0.260, 0.422	0.239, 0.388	0.226, 0.373

Legend: The adjusted odds ratio of gait speed on all-cause 5-years mortality. Gait speed was systematically missing at the study site of Blekinge. Fully conditional specification (FCS), multivariate normal imputation (MVN), and conditional quantile imputation (CQI) were applied based on 100 imputations. Complete Case (CC) analysis was based on the three studies with complete data. The area under the curve was determined for each imputation method and the CC. Adjustments were made for severe disability (ADL), mild disability (IADL), cognitive functional status measured with the Mini-Mental State Examination (MMSE), number of chronic diseases (comorbidities), all-cause mortality (5-years mortality), sex (female), and age (in years).

## DISCUSSION

This simulation set out to assess the performance of three MI strategies for a systematically missing discrete predictor – gait speed – in an IPDMA based on data from SNAC. We compared two established methods, FCS and MVN, and one method based on conditional quantiles (CQI). The results of a large number of replications indicated that the relative bias was less than 1% for the CQI method, whereas it ranged from 2% to 10% for the other two common imputation methods, FCS and MVN. In addition, the results indicate that FCS and MVN show a slightly better precision compared to CQI. Despite differences in performance measures, from a substantive point of view, the differences in estimated odds ratios of gait speed on 5-years mortality were not substantial between the evaluated methods which ranged from 0.54 - 0.57 for the first level of gait speed (0.8-1.2 vs ≤ 0.8 m/s) and 0.29 - 0.33 for the second level of gait speed (>1.2 vs ≤ 0.8 m/s).

The investigated methods FCS and MVN show a relative bias between 2 to 10%. Previous studies comparing MI methods including FCS and/or MVN have shown lower relative biases. [8, 10, 23, 30]. However, none of the previous studies have assessed the MI approaches for a systematically missing discrete predictor in an IPDMA with only four included studies and a common-effect MA. Audigier et al. [8] presented a relatively small bias for the FCS method considering a minimal cluster size of seven. However, the authors used a random-effects MA to evaluate multiple MI strategies. A comparison of results between the two studies has thus to be done with consideration of this major difference. Yet, the somewhat larger bias in this simulation could be potentially explained by a limited size of included studies.

MVN was previously tested on binary variables for sporadically missing data by several studies [23–25] and for continuous variables in longitudinal studies [30], indicating a reasonable performance. Applying MVN to systematically missing discrete data in an IPDMA with a small number of studies in this simulation indicated the largest relative bias compared to the other two approaches. This highlights the challenges of using MVN in this specific context. Our findings suggest that applying MVN to impute systematically discrete data within IPDMA with a small number of studies might be more challenging compared to other contexts in previous studies. Future methodological work should explore broader scenarios and potentially include alternative methods like predictive mean matching for comparison. However, few simulation studies have evaluated these approaches in similar scenarios similar to the one presented in this study.

Continuous efforts are needed to test various imputation approaches in realistic IPDMA scenarios, such as those encountered in aging research shown in the SNAC. The observed lower confidence interval coverage for specific levels of gait speed (77% compared to the expected 95%) indicates potential limitations of MVN in handling systematically missing discrete predictors within IPDMA with only a small number of studies. This is not a criticism in the method itself but rather the use in application in these specific circumstances.

To the best of our knowledge, the method based on conditional quantiles has not yet been investigated in the context of a systematically missing discrete predictor in an IPDMA. One reason for the limited number of applications of CQI could be the lack of implementation in standard statistical software. Still, quantile imputations have been assessed in different contexts before [26] and indicate good performances. Our findings show a small relative bias, yet a larger model-based and empirical SE compared to the other two for the CQI method.

### Strengths and limitations

We investigated one specific scenario of multiple imputation of a systematically missing discrete variable in an IPDMA with only four studies. The simulations were based on unique data from SNAC and have a practical relevance for researchers involved in working with SNAC and similar data. Further, this is one of the first specific simulations that investigate multiple imputation of systematic missing variables in the context of IPDMA with only a small (<5) number of studies. Last, the imputation method based on condition quantiles operated sufficiently well for small IPDMA and should be further explored.

We acknowledge several limitations. First, the simulations in this paper may have restricted generalisability. Our simulations were specifically tailored to the SNAC studies and might perform differently in other scenarios. We chose to relate the simulations to a specific example to have a direct impact on people working with SNAC data. General conclusions are reasonable to be extended to other IPDMA with only a small number of large observational studies. Second, we only explored one specific scenario as a probabilistic sensitivity analysis of multiple imputation for systematically missing data in IPDMA. Future research in this area should be directed towards a more general approach, including scenarios on varying clusters and sample sizes, varying levels for missing predictors, and combining systematically with sporadically missing data. Last, in our simulation settings we assumed a common effect of gait speed on mortality across the four studies. Although the homogeneity of effects can be easily relaxed, it would be very difficult to derive good estimates of variability across studies based on a limited number of studies.

## CONCLUSIONS

Comparing three MI strategies for a systematically missing data on gait speed an IPDMA with four large observational studies from SNAC, we found that the conditional quantile imputation (CQI) approach showed the best performance. Under the characteristics of the IPDMA, the relative bias for the CQI was below 1%, whereas the fully conditional specification (FCS) and multivariate normal (MVN) methods showed biases between 2 and 10%. Moving forward, the CQI strategy should be further evaluated and critically scrutinised to be applied to different contexts.

## Supplementary Material

Supplementary Appendix
