# Supplementary material for: Multiple imputation of systematically missing data on gait speed in the Swedish National Study on Aging and Care
Source: Aging (Albany NY). 2024 Feb 14;16(4):3056–67. doi: 10.18632/aging.205552 (PMC10929840; doi:10.18632/aging.205552)
Supplement: Supplementary Appendix [file aging-16-205552-s001.docx]

**Supplementary Material**

eAppendix A – p.2

eAppendix B – p.3

**eAppendix A**

The matrices for the three studies with complete data (Kungsholmen, Skåne, Nordanstig) are shown below.

$$\Sigma_{1}= \left( \begin{matrix} 0.740 & & & & & & \\ -0.081 & 0.069 & & & & & \\ -0.210 & 0.052 & 0.166 & & & & \\ 0.173 & -0.065 & -0.102 & 0.243 & & & \\ -0.317 & 0.046 & 0.119 & -0.080 & 0.661 & & \\ -0.582 & 0.100 & 0.232 & -0.221 & 0.395 & 1.099 & \\ -0.075 & 0.012 & 0.023 & -0.030 & 0.043 & 0.085 & 0.230 \end{matrix} \right)$$

$$\mu_{1}= \left( 1.188 0.096 0.236 1.785 1.077 1.090 0.649 \right)$$

$$\Sigma_{2}= \left( \begin{matrix} 0.512 & & & & & & \\ -0.038 & 0.041 & & & & & \\ -0.151 & 0.027 & 0.184 & & & & \\ 0.088 & -0.033 & -0.076 & 0.209 & & & \\ -0.174 & 0.019 & 0.082 & -0.013 & 0.450 & & \\ -0.372 & 0.040 & 0.166 & -0.114 & 0.193 & 0.891 & \\ -0.036 & 0.001 & 0.018 & 0.001 & 0.003 & 0.035 & 0.250 \end{matrix} \right)$$

$$\mu_{2}= \left( 0.881 0.077 0.284 1.774 0.482 1.163 0.541 \right)$$

$$\Sigma_{3}= \left( \begin{matrix} 0.361 & & & & & & \\ -0.056 & 0.048 & & & & & \\ -0.086 & 0.030 & 0.183 & & & & \\ 0.086 & 0.026 & -0.037 & 0.246 & & & \\ -0.139 & 0.027 & 0.046 & -0.058 & 0.555 & & \\ -0.297 & 0.047 & 0.090 & -0.129 & 0.252 & 0.845 & \\ -0.041 & 0.007 & -0.018 & -0.002 & 0.034 & 0.043 & 0.248 \end{matrix} \right)$$

$$\mu_{3}=\left( 1.615 0.051 0.241 1.749 1.293 0.751 0.542 \right)$$

The dimensions of the above matrices are reduced by one in the fourth study (Blekinge).

$$\Sigma_{4}= \left( \begin{matrix} 0.048 & & & & & \\ 0.030 & 0.183 & & & & \\ -0.026 & -0.037 & 0.246 & & & \\ 0.027 & 0.046 & -0.058 & 0.555 & & \\ 0.047 & 0.090 & -0.129 & 0.252 & 0.845 & \\ 0.007 & -0.018 & -0.002 & 0.034 & 0.043 & 0.248 \end{matrix} \right)$$

$$\mu_{4}= \left( 0.051 0.241 1.749 1.293 0.751 0.542 \right)$$

**eAppendix B**

We provide details of the three steps SNAC with four studies and a discrete predictor, gait speed, with three levels systematically missing in one of the studies. Of note, the steps can be followed for any number of studies and any levels of the missing predictor.

**Step I: The imputation model**

Consider the discrete predictor $G$, observed in 4-1 studies, with $j=1,\ldots,J$ levels. In our example $J=3$, gait speed is coded as 0, 1, and 2 corresponding to the levels ≤0.8, 0.8-1.2, and >1.2 meters per second, respectively.

Let $\theta_{ij}$ denote the probability that the predictor $G$ is equal to level $j$ conditionally on a set of explanatory variables $Z_{i}$in the *i*-th study $\theta_{ij}|Z_{i} =P(G_{i}=j|Z_{i})$ with $\sum_{j=1}^{J} \theta_{ij}=1$. We consider a multinomial regression model using the lowest level of gait speed (≤0.8 m/s), coded as 0, as referent for contrasting two logits of conditional probabilities:

$\ln\left( \frac{\theta_{ij}|Z_{i}}{\theta_{i0}|Z_{i}} \right)= g_{ij}(Z_{i})$ with $j=1, 2$

The linear predictor for the *i*-th study $g_{ij}(Z_{i})$ includes all the predictors of 5-years mortality (ADL, IADL, MMSE, number of comorbidities, age, and sex) and the 5-years mortality indicator itself. Similar to other imputation approaches such as FCS, the rationale of including several covariates is to increase the plausibility of random missingness conditionally on everything that has been observed.

The linear predictor for the *i*-th study contrasting the conditional logit of the probability of a gait speed 0.8-1.2 m/s ${(G}_{i}=1)$relative to the conditional logit of the probability of gait speed ≤0.8 m/s ${(G}_{i}=0)$ is

$$\ln\left( \frac{\theta_{i1}|Z_{i}}{\theta_{i0}|Z_{i}} \right)= g_{i1}(Z_{i})$$

$$g_{i1}\left( Z_{i} \right)=\gamma_{i1\_0}+\gamma_{i1\_1}\mathrm{ADL}+ \gamma_{i1\_2}IADL+\gamma_{i1\_3}I\left( MMSE=2 \right)+\gamma_{i1\_4}I\left( MMSE=3 \right)+\gamma_{i1\_5} I\left( Comorbidities=2 \right) +\gamma_{i1\_6} I\left( Comorbidities=3 \right)+ \gamma_{i1\_7} I\left( Age=2 \right)+\gamma_{i1\_8} I\left( Age=3 \right)+ \gamma_{i1\_9} I\left( Age=4 \right)+\gamma_{i1\_10}\left( \mathrm{Sex} \right)+\gamma_{i1\_11}(Mortality)$$

Similarly, the linear predictor for the *i*-th study contrasting the conditional logit of the probability of a gait speed >1.2 m/s ${(G}_{i}=2)$relative to the conditional logit of the probability of gait speed ≤0.8 m/s ${(G}_{i}=0)$ is

$$\ln\left( \frac{\theta_{i2}|Z_{i}}{\theta_{i0}|Z_{i}} \right)= g_{i2}(Z_{i})$$

$$g_{i2}\left( Z_{i} \right)=\gamma_{i2\_0}+\gamma_{i2\_1}\mathrm{ADL}+ \gamma_{i2\_2}IADL+\gamma_{i2\_3}I\left( MMSE=2 \right)+\gamma_{i2\_4}I\left( MMSE=3 \right)+\gamma_{i2\_5} I\left( Comorbidities=2 \right) +\gamma_{i2\_6} I\left( Comorbidities=3 \right)+ \gamma_{i2\_7} I\left( Age=2 \right)+\gamma_{i2\_8} I\left( Age=3 \right)+ \gamma_{i2\_9} I\left( Age=4 \right)+\gamma_{i2\_10}\left( \mathrm{Sex} \right)+\gamma_{i2\_11}(Mortality)$$

Estimates $\hat{\gamma}_{i}$ of the $(11+1)\times2=24$ parameters $\gamma_{i}$ in $g_{i1}\left( Z_{i} \right)$ and $g_{i2}\left( Z_{i} \right)$ are obtained using the maximum likelihood method separately for each study (*i* = 1, 2, 3) with complete information (Kungsholmen, Skåne, Nordanstig).

**Step II: Predict conditional probabilities**

All estimates $\hat{\gamma}_{i}$ in $g_{i1}\left( Z_{i} \right)$ of $g_{i2}\left( Z_{i} \right)$ are combined across the three studies with complete information (Kungsholmen, Skåne, Nordanstig) using the inverse-variance method

$$\bar{\gamma}=\frac{\sum_{i=1}^{3} w_{i}\hat{\gamma}_{i}}{\sum_{i=1}^{I} w_{i}}$$

with weight equal to one divided by the estimated variance to obtain two average linear predictors $\bar{g}_{1}\left( Z_{i} \right)$ and $\bar{g}_{2}\left( Z_{i} \right)$ as follows

$$\bar{g}_{1}\left( Z_{i} \right)=\bar{\gamma}_{1\_0}+\bar{\gamma}_{i1\_1}\mathrm{ADL}+ \bar{\gamma}_{1\_2}IADL+\bar{\gamma}_{1\_3}I\left( MMSE=2 \right)+\bar{\gamma}_{1\_4}I\left( MMSE=3 \right)+\bar{\gamma}_{1\_5} I\left( Comorbidities=2 \right) +\bar{\gamma}_{1\_6} I\left( Comorbidities=3 \right)+ \bar{\gamma}_{1\_7} I\left( Age=2 \right)+\bar{\gamma}_{1\_8} I\left( Age=3 \right)+ \bar{\gamma}_{1\_9} I\left( Age=4 \right)+\bar{\gamma}_{1\_10}\left( \mathrm{Sex} \right)+\bar{\gamma}_{1_{11}}(Mortality)$$

$$\bar{g}_{2}\left( Z_{i} \right)=\bar{\gamma}_{2\_0}+\bar{\gamma}_{2\_1}\mathrm{ADL}+ \bar{\gamma}_{2\_2}IADL+\bar{\gamma}_{2\_3}I\left( MMSE=2 \right)+\bar{\gamma}_{2\_4}I\left( MMSE=3 \right)+\bar{\gamma}_{2\_5} I\left( Comorbidities=2 \right) +\bar{\gamma}_{2\_6} I\left( Comorbidities=3 \right)+ \bar{\gamma}_{2\_7} I\left( Age=2 \right)+\bar{\gamma}_{2\_8} I\left( Age=3 \right)+ \bar{\gamma}_{2\_9} I\left( Age=4 \right)+\bar{\gamma}_{2\_10}\left( \mathrm{Sex} \right)+\bar{\gamma}_{2_{11}}(Mortality)$$

The predicted conditional probability of falling into the three levels of the systematic missing variable, gait speed, for any individual in the fourth study (Blekinge) is obtained by using the observed variables $Z_{4}$ in such study

$$\hat{\theta}_{0}=\hat{P}\left( G_{4}=0 | Z_{4} \right)=\frac{1}{1+e^{\bar{g}_{1}\left( Z_{4} \right)}+e^{\bar{g}_{2}\left( Z_{4} \right)}}$$

$$\hat{\theta}_{1}=\hat{P}\left( G_{4}=1 | Z_{4} \right)=\frac{e^{\bar{g}_{1}\left( Z_{4} \right)}}{1+e^{\bar{g}_{1}\left( Z_{4} \right)}+e^{\bar{g}_{2}\left( Z_{4} \right)}}$$

$$\hat{\theta}_{2}=\hat{P}\left( G_{4}=2 | Z_{4} \right)=\frac{e^{\bar{g}_{2}\left( Z_{4} \right)}}{1+e^{\bar{g}_{1}\left( Z_{4} \right)}+e^{\bar{g}_{2}\left( Z_{4} \right)}}$$

where the sum of the predicted probabilities for each individual in the fourth study is

$$\sum_{j=1}^{J} \hat{\theta}_{j}=1$$

**Step III: Impute values for the predictor with systematic missing**

Let $G_{4}^{I}$ denote the imputed variable representing gait speed, a predictor of 5-years mortality, that is systematically missing in the fourth study. The inverse cumulative distribution function method is used to assign a value to $G_{4}^{I}$. The predicted cumulative distribution function, varying across individuals according to the observed variables in $Z_{4}$, says that

$${P(G}_{4}^{I}\leq0|Z_{4})= \hat{\theta}_{0}$$

$${P(G}_{4}^{I}\leq1|Z_{4})= \hat{\theta}_{0}+\hat{\theta}_{1}$$

$${P(G}_{4}^{I}\leq2|Z_{4})= \hat{\theta}_{0}+\hat{\theta}_{1}+\hat{\theta}_{2}$$

A value for $G_{4}^{I}$ is assigned by inverting a cumulative distribution function of a uniform distribution over the interval 0 to 1. The imputed value $G_{4}^{I}$ is the quantile, $Q$, corresponding to the predicted cumulative conditional probabilities estimated in the previous step where $U$ is randomly drawn from a $\mathrm{Uniform}(0,1)$

$$2= Q_{\hat{\theta}_{0}+\hat{\theta}_{1}+\hat{\theta}_{2}}\left( U \right)$$

$$1=Q_{\hat{\theta}_{0}+\hat{\theta}_{1}}\left( U \right)$$

$$0=Q_{\hat{\theta}_{0}}\left( U \right)$$
